# Motivating household water conservation: A field experiment in Singapore

**DOI:** 10.1371/journal.pone.0211891

**Published:** 2019-03-20

**Authors:** Lorenz Goette, Ching Leong, Neng Qian

**Affiliations:** 1 Department of Economics, National University of Singapore, Singapore, Singapore; 2 Institute of Water Policy, Lee Kuan Yew School of Public Policy, National University of Singapore, Singapore, Singapore; Kansas State University, UNITED STATES

## Abstract

We test and compare different incentives in motivating water conservation using a randomized controlled trial. In a field experiment carried out with Singaporean households, regular feedback was given, with informative, normative and monetary incentives provided to different groups. Evidence shows that all households saved an average of 4 Litres of water per person per day, with no difference in treatment effect found across various groups. Perhaps unsurprisingly, the water saving effect is also found to be more significant with high baseline users, who saved up to 5.9 Litres per person per day. High baseline households also respond more positively to the non-monetary incentives.

## 1 Introduction

Apart from being a vital human need, water is also an economic resource critical for socio-economic development. Today, the problems of water scarcity and water stress that confront many societies have been aggravated by the rapid growth of population and climate change. According to the United Nations, water use has been growing at more than twice the rate of population increase over the last century.

Given the increasing demand for water and the uncertain future in supply, there is a pressing need for policy makers to engage the public in water conservation. As of today, there are wide applications of demand side management tools in different parts of the world. Typical policy tools include financial, technological, and educational interventions [1]. Adjustment of water tariffs and taxes, introducing and installation of water efficient devices, water rationing and mandatory allocation policies as well as educational campaigns are common examples.

While there are extensive studies using econometric models to evaluate the effectiveness of different interventions, the results are contentious. For example, in general the residential water demand is found to be price inelastic [2–4]. Other literature has shown that although pricing tools may affect demand in the short run, there could be behaviors counteracting the shock and water demand may rebound in the long run [5, 6]. Similar results are found for technological interventions, where the policy makers offers new resource-efficient technology or devices, or even mandate such engineering solutions [7]. Another strand of literature focus on the socio-psychology of water conservation, identifying the key factors that drive water-saving behavior, such as attitudes, beliefs, habits, etc [8–10].

In recent years, randomized controlled trials (RCT) have been widely used to test different behavioral hypotheses as well as various policy interventions, in the domain of household resource conservation. Particularly relevant are energy and water conservation programs, where studies show that norm based information and feedback have positive effect in nudging conservative behavior; while purely educational or campaign messages have only limited impact [11–14]. More recently, RCT works are extended to more tuned treatments and interaction of interventions, such as frequency of feedback/information provision, interaction of social comparison with existing conservation programs; various results are established on identifying the causal chanels as well as how more effective interventions can be designed [15–17].

Given the existing evidence obtained in different contexts, our research design puts together informative, normative and monetary incentives for water conservation, in a single field experiment set to compare the irrelative effectiveness.

We used a randomized controlled trial in 1,000 Singaporean households to test the water conservation effect of campaign message, normative feedback and monetary prize for saving water. In a 3-month period, we found that all treated households achieved on average more than 4 Litres of water per person per day compared to control group households; however, no difference was found across treatments. Further, the heterogeneous treatment effect was evident, in the sense that high baseline users responded much more than low baseline users, which is consistent with literature.

## 2 The experiment

### 2.1 Implementation and measurement

**Fig 1:**
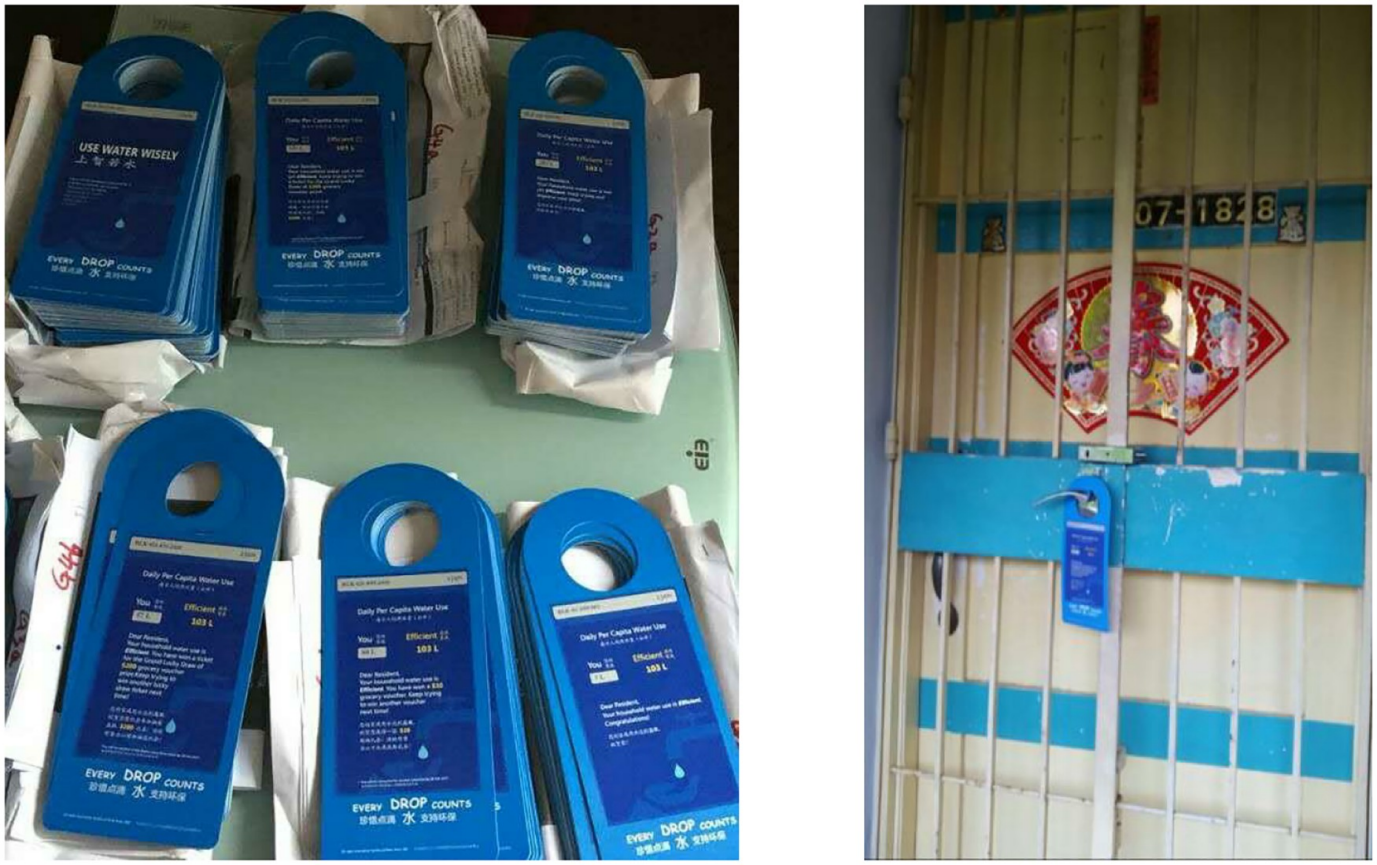
Illustration of door hangers.

This study received the Institutional Review Board of National University of Singapore, Approval Number “NUS 2949”. Written consent was obtained from participants. In this study, we experimented with residential households on their water consumption and conservation behavior at home. Since the local water utility company provides only bimonthly meter reading, we sent our own personnel to read residential water meters and computed the consumptions in much more frequently. Combined with the household survey data we were able to compute the LPCD (litres per capita per day) for each household as the measure for household water consumption. To provide information and feedback to the households, we used printed leaflets in the form of door hangers (Fig 1), which helped to achieve frequent and in-time communications.

The experiment consists of three phases: recruitment phase, treatment phase and post-treatment phase. In the recruitment phase from late November to 10 December of 2016, a survey company was hired to engage participating households clustered in several HDB blocks in Ang Mo Kio area of Singapore. HDB flats are the public housing governed and developed by the Singapore government, which are home to more than 80% of the local resident population. The housing standard, estate management as well as transactions are strictly regulated by the government. We adopted such a sampling strategy in order to minimize heterogeneity across households. During this phase, eligible households (residential households of either Singaporeans or Permanent Residents) completed a survey questionnaire as well as consented to participate in a research regarding water consumption at home. The survey company also took an initial (first) water meter reading for all 1000 households in this span of two weeks.

After the recruitment, we collated the household address and size (number of people living in the flat) and carried out the randomization practice. The recruited households were randomly assigned to 5 groups, 200 each in one control and four treatment groups. The sample size and number of treatment arms were determined *ex ante* based on the power analysis using figures from the literature. We used the expected effect size of 5.25 litres, a standard deviation of 20, a significance level of 5%, and desired power of 80% to compute the sample size for each group to be around 220. The literature of nudge on household water conservation finds the average water saving effect to be around 2-5% [15, 18–20]; the Singapore average water usage per capital per day (LPCD) is 150 litres so we expect the effect size to be around 5.25 liters. From the work of Agarwal et al. [21], the standard deviation of Singapore HDB household water use (LPCD) is 64. Assuming the intra-household monthly correlation of water use to be 0.85, we estimated the standard deviation of the difference measure of average water use LPCD (current period water consumption differenced by the baseline period water consumption) to be 20.

On 17 December 2016, the treatment phase was strated with the first batch of leaflets distributed to 800 treatment group households. On the same day, a second meter reading was taken by the survey company for all households, and the baseline water usage data was hence constructed based on the consumption before treatment. In the treatment phase, we took meter reading once every four days (except for one round during the Christmas holiday which took a 12-day gap) for all households, for a total of six rounds; and distributed leaflets to treatment group households on the same day of meter reading since the second round, for a total of five batches. On 18 January 2017, the treatment group households received the last batch of leaflets and were notified of the completion of the program.

In the post-treatment phase, the water consumptions for all households were tracked in a six-week period. In the first two weeks, water meter readings were taken every four days (expect for one round during the Chinese New Year holiday which took a 6-day gap), for a total of three rounds. In the next four weeks, water meter readings were taken biweekly, for another two rounds.

**Fig 2:**
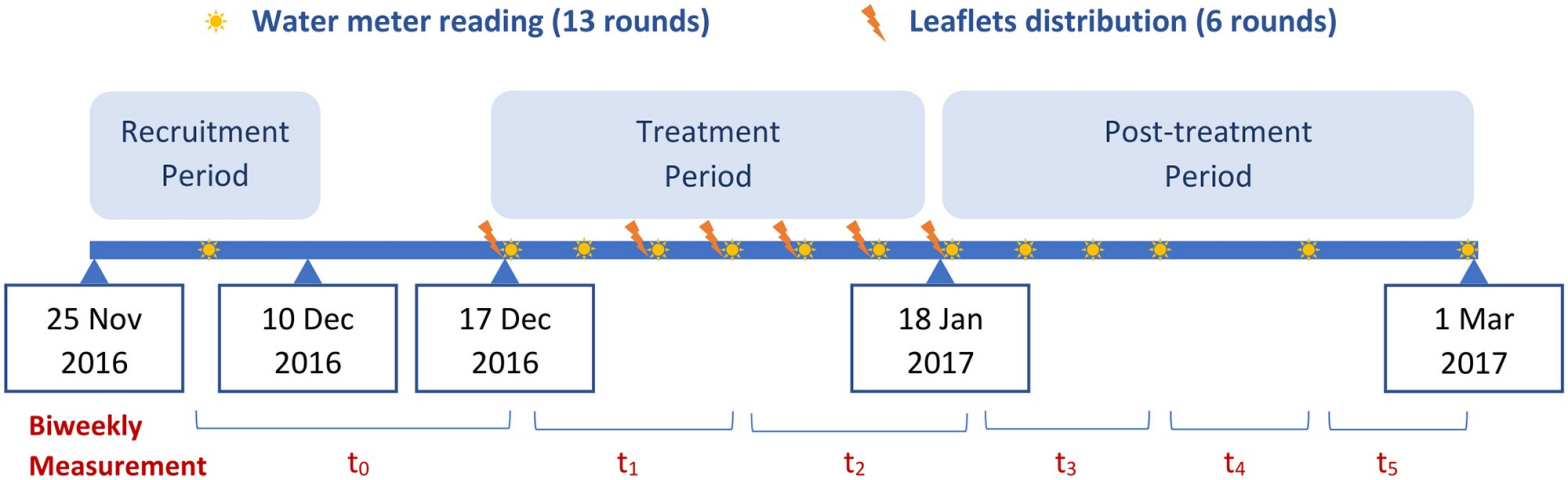
Timeline of the study.

The timeline and measurement of the experiment are illustrated in Fig 2.

In total, we collected 12 LPCD observations of household water consumption, including a baseline usage, six treatment-phase usages and five post-treatment usages. Due to the irregular data collection frequency, we reconstructed the raw data to an approximately biweekly observation data-set. Analysis using the original dataset does not alter the main conclusion of the paper. Biweekly LPCD measure *y*_*it*_ was computed for each household *i* with a total of 6 observations (*t* ∈ {0, 1, …, 5}), i.e. a baseline usage, two treatment-phase usages and three post-treatment usages. As shown in Fig 2, the baseline period *t*_0_ is from one to three weeks; *t*_1_, *t*_2_ and *t*_3_ are of 16-day gap; *t*_4_ and *t*_5_ are of two-week period. All the analyses would be carried out using this revised data structure.

### 2.2 Experimental conditions

We have five experimental conditions, a control condition and four treatment conditions. For the control group, only water meter readings were implemented without any information communicated or feedback. For the treated group, different types of treatment information in both English and Chinese language were provided via the leaflets addressed to individual households. The detailed illustration of sample leaflets could be found in S1 and S2 Figs.

On the back side of all the leaflets, six ‘Water Saving Tips’ were printed. These tips were extracted from the local water authority (Public Utility Board, in short PUB), and all of them are common and easy ways to practice water conservation at home in the local context. On the front side, specific messages corresponding to different treatment conditions are illustrated below:

1. **Campaign group**. A post-like campaign message was designed, with the headline ‘Use Water Wisely’ and a piece of general information why one should use water wisely, reading ‘Every society shares the responsibility to promote sustainable use of water. The future is in our hands.’
2. **Feedback group**. Households received feedback message including their own water consumption in LPCD for the last measurement period, and an ‘Efficient LPCD’ benchmark. The *Efficient LPCD* is calculated as the average water usage by the lowest 50% water using households in the control group for the same period. The formula is according to the current municipal water bill regulated by PUB. However we did not distinguish flat types within the control group. Households were also explicitly identified as ‘Efficient’ or ‘not yet Efficient’.
3. **Rebate group**. Besides the feedback message, the ‘Efficient’ households would win a $10 grocery voucher as a prize.
4. **Lucky draw group**. Besides the feedback message, the ‘Efficient’ households would get a chance to win $200 grocery voucher in a lucky draw. There will be two winners for each round.

### 2.3 Sample and descriptive statistics

During the recruitment phase, the surveyors went to the HDB blocks and recruited the participating households door by door. All the households living in the blocks were visited, from top floor downwards, until the target number of households were achieved. The suveyors had to make sure that the participating individual who answered the questionnaire should be 18 years old above. Eligible households were given a $10 grocery voucher upon completion of a 15-min short survey, and consented to participate in the research program. They were informed that their water meters were to be read and they might receive information leaflets in the next two months, but no further face-to-face visit would take place. 1000 households from 112 blocks were recruited and randomly assigned to five groups. In the treatment phase, 14 households called in to withdraw from participation, leaving a raw sample size of 986. After data cleaning, 87 households were removed due to measurement error such as the lack of baseline water consumption, abnormal meter readings, more than half of readings to be zero, etc., resulting in a final effective sample size of 899 households.

The short survey collected socio-demographic data (household size, income, housing situation, respondent’s age, education, income, etc.), utility bill payment modes, perceptions on water conservation, etc. However, only household address and size information were collated for randomization, due to the tight schedule. Table 1 shows the summary statistics of the key variables by group for the full sample, with randomization checks. Except for the baseline water use (LPCD) which is slightly unbalanced, F-tests show that randomization produced balance between groups on the key observable characteristics.

**Table 1:** Summary statistics and randomization checks.

Variable	Full sample	Control group	Campaign group	Feedback group	Rebate group	Lucky draw group	F test (p-value)
*N* =	899	184	175	173	185	182	
Mean of household size (members)	3.456 (1.66)	3.424 (1.58)	3.434 (1.67)	3.532 (1.61)	3.438 (1.63)	3.456 (1.81)	0.12 (0.9753)
Mean of baseline water use (LPCD)	165.68 (96.27)	176.64 (111.01)	158.50 (77.87)	156.62 (86.68)	159.45 (87.99)	176.44 (110.77)	1.99 (0.0935)
Median of baseline water use (LPCD)	145.93	157.75	145.3	140.77	139.75	145.19	
Gender (fraction of female)	0.54 (0.50)	0.61 (0.48)	0.53 (0.50)	0.53 (0.50)	0.54 (0.50)	0.49 (0.50)	1.65 (0.1601)
Age (of respondent)	56.61 (14.74)	57.01 (14.81)	56.76 (15.17)	57.30 (13.98)	55.04 (14.91)	56.97 (14.67)	0.68 (0.6071)
Annual Household income (Singapore Dollar)	30874 (32658)	28900 (29117)	27717 (30112)	33541 (38072)	34426 (35439)	29793 (29864)	0.92 (0.4501)
Citizenship (Singaporean = 1)	0.91 (0.28)	0.92 (0.27)	0.93 (0.25)	0.90 (0.29)	0.90 (0.29)	0.90 (0.29)	0.47 (0.7609)
Ownership of flat (= 1)	0.82 (0.37)	0.81 (0.38)	0.86 (0.33)	0.79 (0.40)	0.80 (0.40)	0.86 (0.34)	1.56 (0.1833)
Flat type (no. of rooms)	3.14 (0.94)	3.09 (0.95)	3.17 (0.91)	3.16 (0.96)	3.07 (0.97)	3.18 (0.90)	0.52 (0.7237)
No. of helpers at home	0.11 (0.33)	0.07 (0.25)	0.11 (0.35)	0.09 (0.29)	0.13 (0.34)	0.14 (0.40)	1.67 (0.1552)
No. of children at home (under 18 yrs)	0.63 (1.04)	0.58 (1.09)	0.57 (0.89)	0.63 (0.98)	0.63 (1.07)	0.72 (1.15)	0.56 (0.6930)
Water conservation practice † (Scale 1-5)	4.01 (0.67)	4.01 (0.63)	4.02 (0.65)	4.02 (0.61)	4.01 (0.74)	3.97 (0.72)	0.20 (0.94)

*Note*: The table reports the group means of key socio-demographic variables and the water consumption for the baseline period. The standard deviations are included in parentheses. The last column illustrates the test statistics of the randomization checks performed on these key variables. A two-sided ANOVA was conducted to verify whether the randomization has successfully produced balance on observable key characteristics between the five experimental conditions before the treatment.

^†^ The statement “My household has done adequately in conserving water” was asked in a 1-5 scale of agreement in the survey questionnaire, to proxy the self-reported water conservation practice.

## 3 Results

We implemented a difference-in-difference analysis on the biweekly water consumption measurement *y*_*it*_. A difference term $\Delta_{y_{it}}=y_{it}-y_{it_{0}}$ (*t* = 1, 2, …, 5) was constructed, which measures the household *i*’s change in water use in a particular observation period compared to the water consumption of baseline period. Five observations of $\Delta_{y_{it}}$ were obtained, with the first two in treatment period while the remaining three in post-treatment period.

### 3.1 The overall treatment effect

**Fig 3:**
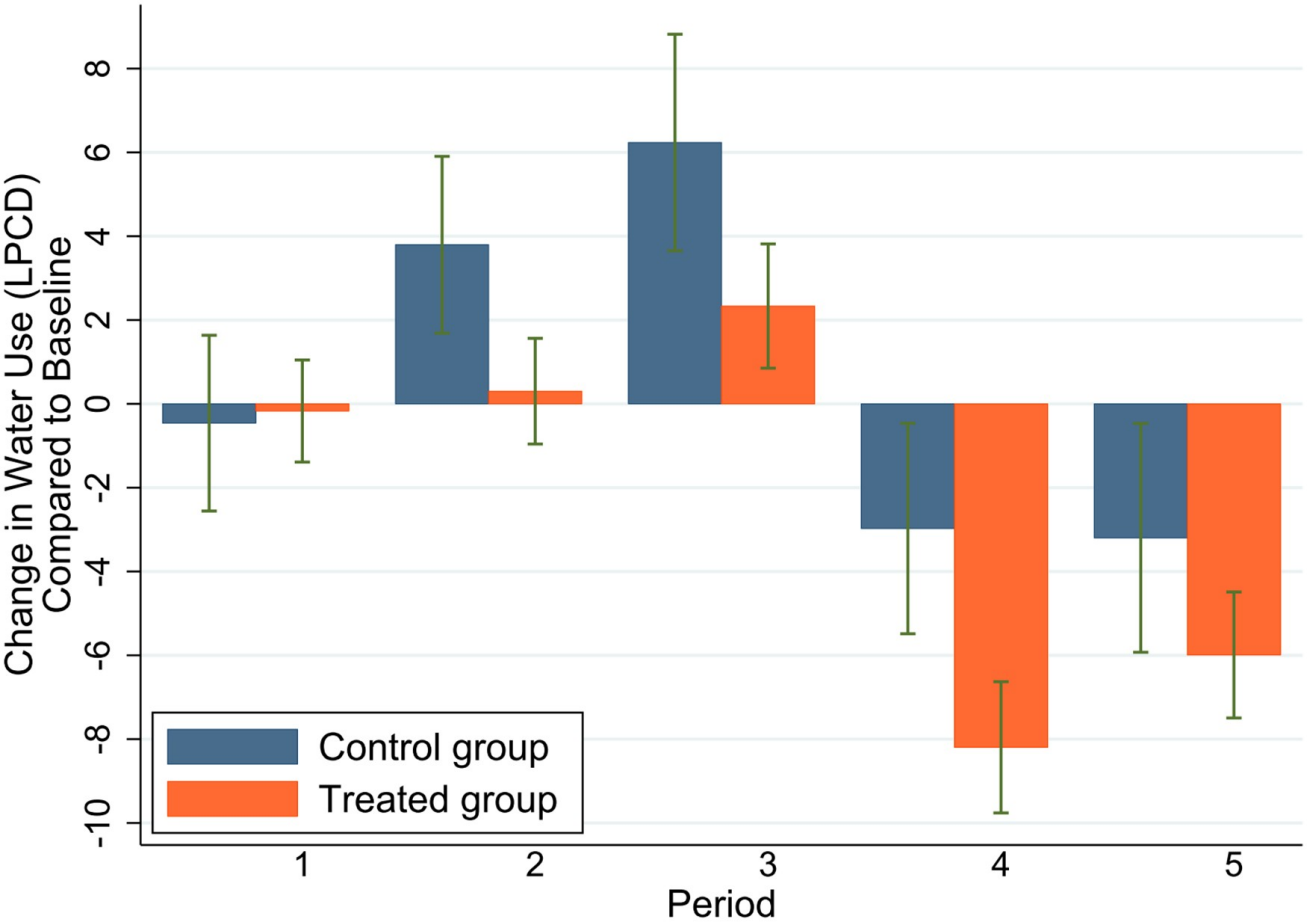
Difference-in-difference estimate of the overall treatment effect by periods. *Note*: Each bar indicates the average change of LPCD within households in water use compared to the baseline period. Error bars mean +/- SEM. This applies to all the bar charts in the remaining sections.

We first look into the overall treatment effect, i.e. whether there is any water conservation effect purely out of being treated, without differentiating the individual treatments. In this sense, we group the households from all four treatment conditions as one single group—the ‘treated group’. Fig 3 shows the difference-in-difference estimates of the overall treatment effect for each period. For both control and treated groups, we calculated the group mean of the change in water use $\Delta_{y_{it}}$; the comparison of such means between groups allows us to gauge the overall treatment effect. The figure shows an increasing trend in the first three periods compared to the baseline period, and a reduction in water use in the last two periods, for both control and treatment group households. Except for the first period, the magnitude of $\Delta_{y_{it}}$ of the treated group is always below that of the control group. That means, for periods when water use increased, the incremental amount is smaller in the treated group; for periods when water use decreased, the reduction is larger. The standard-error bars around the means indicate that the difference between the treatment and the control groups is significant.

Formally, we estimated the model

$$\begin{array}{c} \Delta_{y_{it}}=\alpha +\beta_{bl}x_{i}+\beta_{T}T_{i}+d_{t}+\epsilon_{i}. \end{array}$$ (1)

The dependent variable $\Delta_{y_{it}}$ is the change in water use for household *i* at period *t*, which is defined earlier. The baseline water consumption *x*_*i*_ (which is essentially $y_{it_{0}}$) for each household is included to account for possible variations brought by difference in baseline usage. The treatment dummy *T*_*i*_ is 0 for control group and 1 for all treated group households. A time fixed effect *d*_*t*_ is included to capture the time trends of water consumption for all households. The error term *ϵ*_*i*_ captures any unmodeled effects that are orthogonal to our treatment conditions by virtue of randomization. Hence, the estimate of *β*_*T*_ would indicate the overall treatment effect, i.e. how the average change of water consumption from baseline period by the treated group differs from that by the control group households. To differentiate the overall treatment effect by treatment periods and post-treatment periods, we estimated the same model (1) in particular time frames: for all periods, for treatment periods *t*_1_ and *t*_2_, and for post-treatment periods *t*_3_ to *t*_5_. Table 2 presents the results.

**Table 2:** The overall treatment effects.

	All periods (*t* = 1 ∼ 5)	Treatment periods (*t* = 1 ∼ 2)	Post-treatment periods (*t* = 3 ∼ 5)
	(1)	(2)	(3)
Treatment: all treatment groups. (= 1)	−4.21** (2.15)	−2.35 (2.06)	−5.44** (2.51)
Baseline water usage in LPCD (*x*_*i*_)	−0.086*** (0.015)	−0.054*** (0.012)	−0.107*** (0.02)
Constant	3.116 (1.967)	1.642 ((1.830))	7.461*** (2.289)
R squared	0.062	0.027	0.081
Observations	4495	1798	2697

*Note*: The table illustrates the overall treatment effects for the whole observation periods, treatment periods and post-treatment periods, controlling for the household baseline water use and time fixed effects. Standard errors are adjusted for clustering at the household level, reported in the parentheses.

*, ** and *** indicate the significance level of 10%, 5% and 1% respectively.

The first column of Table 2 shows the overall treatment effect across all the observation periods (*t*_1_ to *t*_5_). The results confirm that there is water conservation effect of being treated: those who received regular leaflets containing water saving tips, with or without feedback or incentives, used 4.21 Litres less water per capita per day than the control group did. The second and third columns display the estimates for different periods, which precisely reflects the impression of Fig 3. The water conservation effect is initially mild (2.35 Litres) in the treatment periods *t*_1_ and *t*_2_, but becomes most significant (5.44 Litres) in the post-treatment periods *t*_3_ and *t*_5_, in terms of both effect size and significance. The treatment seems to take effect with some lag, and the effect does not vanish even after the treatment has been removed for several observation periods.

### 3.2 Treatment effects by experimental conditions

**Fig 4:**
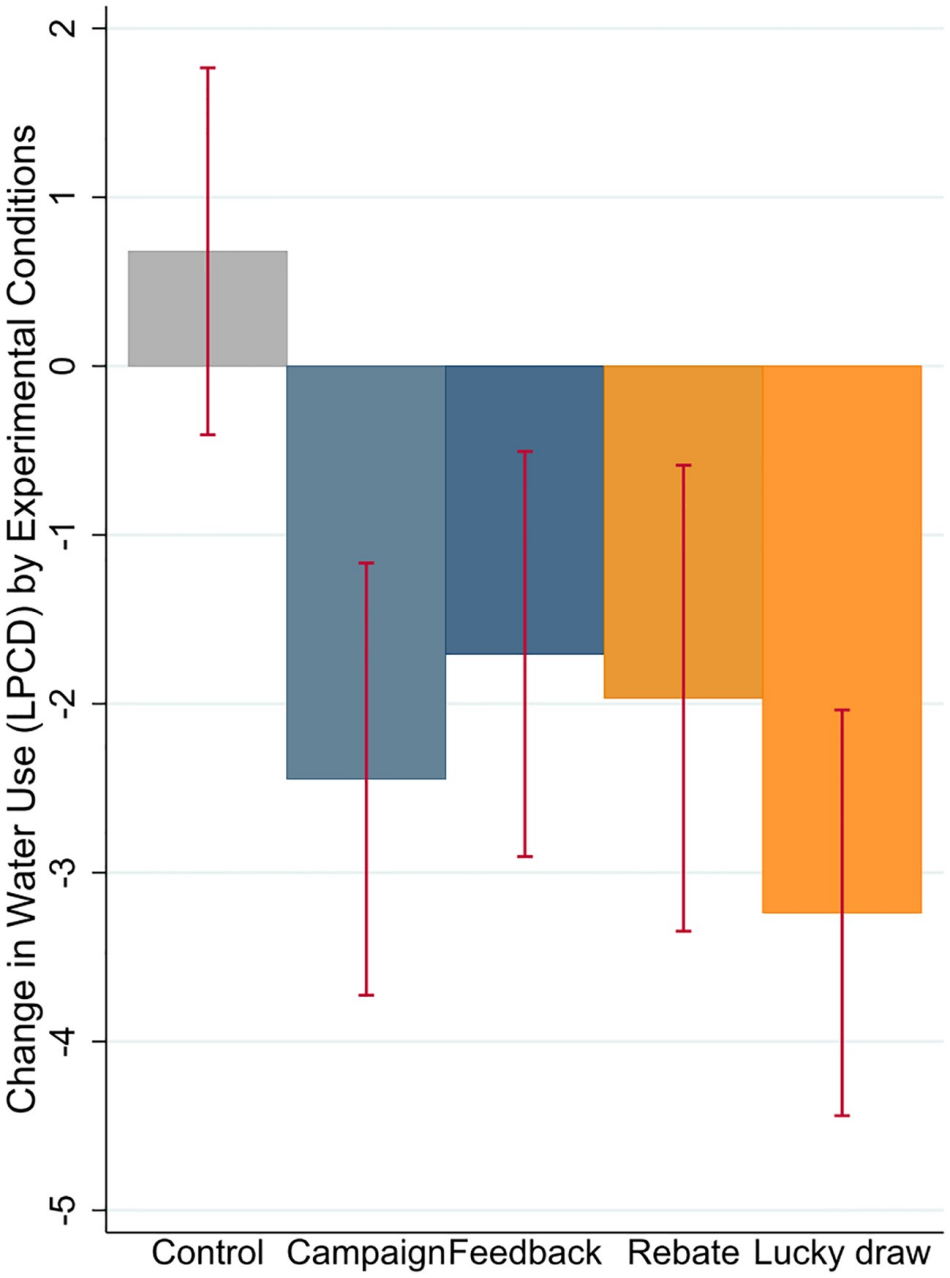
Treatment effect by conditions.

In this section, we examine the treatment effects by the four different experimental conditions. Fig 4 illustrates the group means and standard errors of the change in water use compared to baseline period. It can be seen that all four treatment groups achieve some reduction in water use compared to the control group; however there is no significant difference in the effect sizes across the four treatment conditions. Such observation is tested in the formal estimation.

We estimated the model

$$\begin{array}{c} \Delta_{y_{it}}=\alpha +\beta_{bl}x_{i}+\beta_{1}T_{1i}+\beta_{2}T_{2i}+\beta_{3}T_{3i}+\beta_{4}T_{4i}+d_{t}+\epsilon_{i}. \end{array}$$ (2)

The dependent variable $\Delta_{y_{it}}$ and control variables *x*_*i*_ and *d*_*t*_ are the same as defined in Model (1). The four treatment dummies, *T*_1*i*_ to *T*_4*i*_, correspond to the experimental conditions of ‘Campaign’, ‘Feedback’, ‘Rebate’ and ‘Lucky draw’ respectively for each household. Hence, the parameters *β*_1_—*β*_4_ indicate the different treatment effects: how the average change of water use differ between each treatment group and the control group. Table 3 shows the results.

**Table 3:** Estimates of Model (2).

	Estimates of Model 2
Campaign (*T*_1_ = 1)	−4.68* (2.82)
Feedback (*T*_2_ = 1)	−4.11 (2.71)
Rebate (*T*_3_ = 1)	−4.12 (2.94)
Lucky draw (*T*_4_ = 1)	−3.94 (2.76)
Baseline	−0.086*** (0.016)
Constant	17.35*** (3.08)
F test: all treatments have the same effect.	*p* = 0.9948
R squared	0.0619
Observations	4495

*Note*: The table displays the treatment effects by the four experimental conditions, controlling for the household baseline water use and time fixed effects. Standard errors are adjusted for clustering at the household level, reported in the parentheses.

*, ** and *** indicate the significance level of 10%, 5% and 1% respectively.

We can read from the table that each treatment achieves a conservation effect of around 4 Litres per capita per day compared to the control group, with a standard error of around 2.8 Litres. Given the effect size and standard errors, the treatment effects are not significant except for the ‘Campaign’ group, which is significant at the 10% level. The F-test statistics also confirms that no significant difference is detected among the four groups, in terms of treatment effect.

Such results suggest that receiving ‘Water Saving Tips’ did make a difference, however neither normative incentives (‘Feedback’) nor monetary incentives (‘Rebate’ or ‘Lucky draw’) brought in significantly different impact on water saving. In other words, campaign-only message has almost similar effect to that of feedback and/or monetary incentives.

One possible reason for the similar treatment effects might be the particular information in the feedback that was provided. For all the treated households but the ‘Campaign’ group, an **“Efficient”** water consumption benchmark is printed on the leaflets, besides their own household water use. This benchmark is computed as the average water consumption of the lowest 50% water users, which is according to the official bill of the local water utility. However, the “Efficient” benchmark is around 100 Litres per capita per day, almost equivalent to the 1st quartile of water consumption for all the sample households. Given the average LPCD of 165 Litres, it seems too high for most households to achieve this benchmark. Residents might find it extremely difficult to achieve 100 Litres, and thus have no motivation to respond to such additional incentives.

Hence, there is insufficient evidence from this study to determine whether normative or monetary incentives have additional or different conservation effects, on top of the effect from campaign message with water saving tips.

### 3.3 Heterogeneity in the responses by low and high baseline households

In this section, we investigate the heterogeneity in treatment effects by different household groups, which is also noted by other scholars in the literature [18].

**Fig 5:**
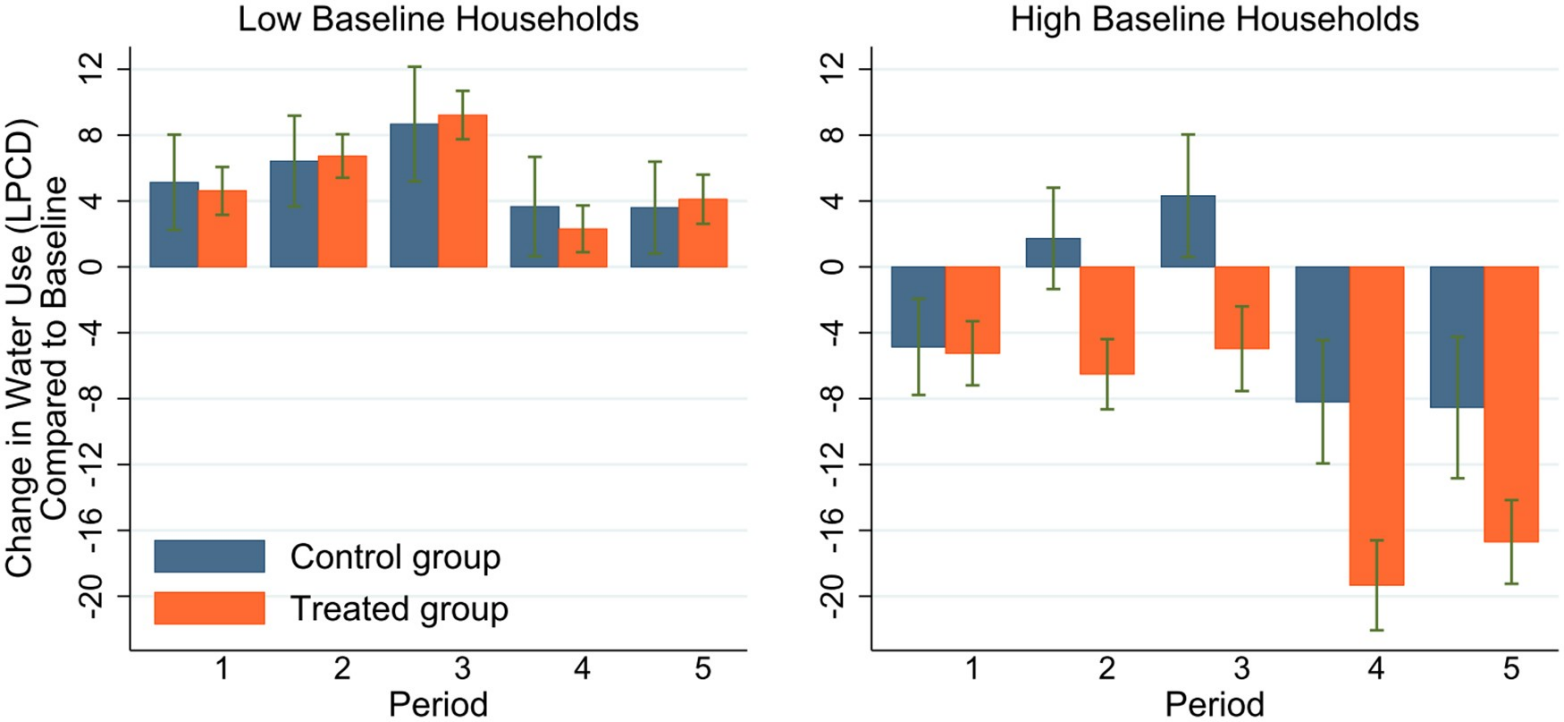
Heterogeneity in treatment effects.

We divide the treated group into two subgroups: low and high baseline households, which refer to those with the baseline period LPCD below and above the median baseline water usage of the sample respectively. Fig 5 illustrates the difference in treatment effects by the two subgroups. It clearly shows that high baseline households respond more to the treatment, by saving much more water than the low baseline households. In fact, there is almost no treatment effect with low baseline households.

**Fig 6:**
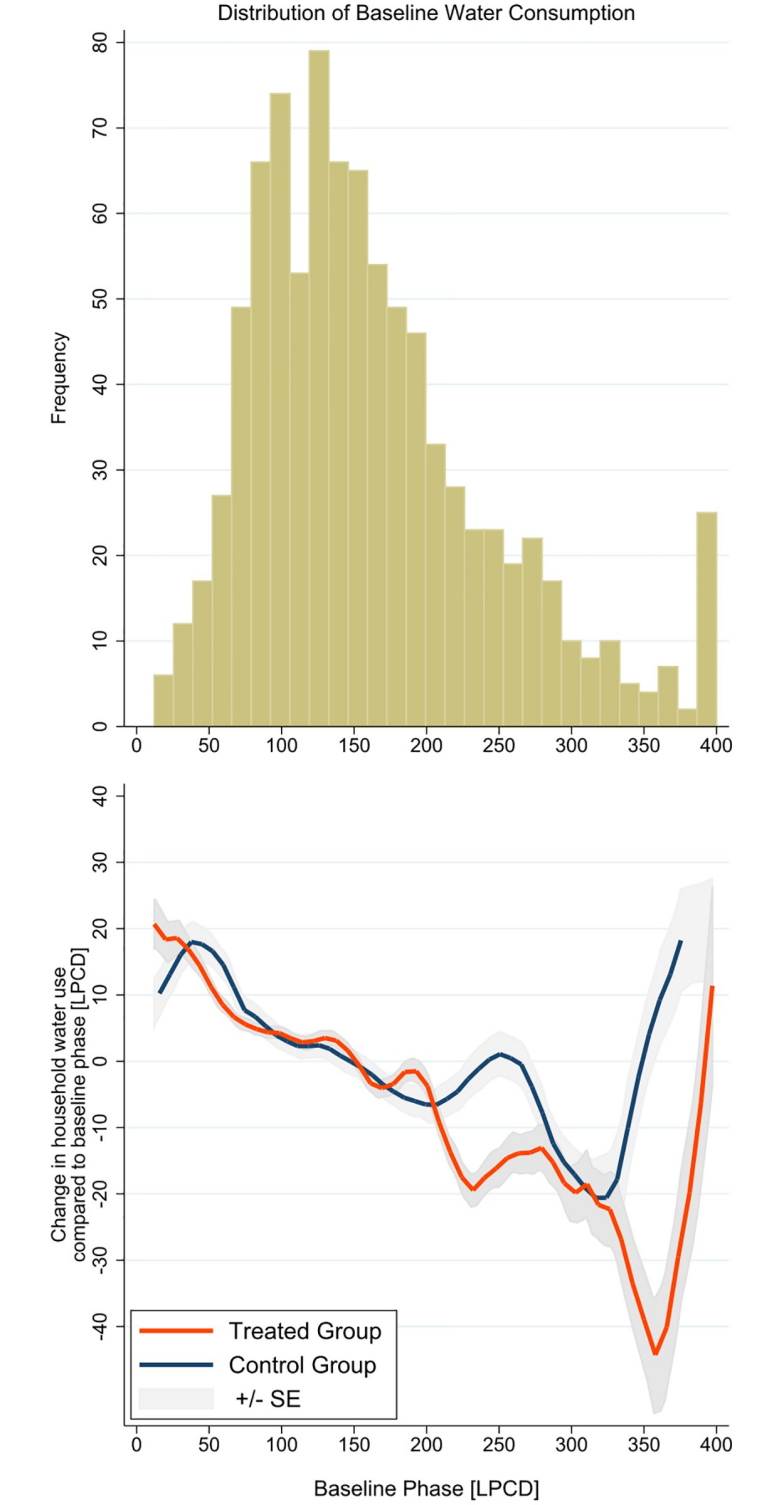
Nonparametric estimate of the overall treatment effect.

The observation can also be shown by the non-parametric estimation against the distribution of households according to the baseline consumption. The top panel illustrates the distribution of households by the baseline water usage of Fig 6; while the bottom panel plots the change in water use by control and treated households against baseline water usage. As the baseline phase water consumption gets higher, the gap between the two groups becomes larger. In other words, the treatment effect is more significant with high baseline households. Such effect is most significant for the households with baseline usage between 200 to 300 in LPCD.

Formally, we estimate the model

$$\Delta_{y_{it}}=\alpha +\beta_{bl}x_{i}+\beta_{T}T_{i}+\gamma_{T}T_{i}\cdot x_{i}+d_{t}+\epsilon_{i}.$$ (3)

$\Delta_{y_{it}}$, *x*_*i*_, *T*_*i*_ and *d*_*t*_ are as defined earlier. *T*_*i*_ ⋅ *x*_*i*_ is the interaction of treatment dummy and baseline usage. The parameter *β*_*T*_ captures the treatment effect for 0 baseline households, and *γ*_*T*_ indicates how the treatment effect changes with the baseline water usage. By allowing the treatment effect to vary with baseline water usage, this model can identify the heterogeneity in the overall treatment effect, by different baseline usage households.

A counterpart of Model (3) is

$$\begin{array}{c} \begin{array}{cc} \Delta_{y_{it}}=\alpha & +\beta_{bl}x_{i}+\beta_{1}T_{1i}+\beta_{2}T_{2i}+\beta_{3}T_{3i}+\beta_{4}T_{4i}\\ & +\gamma_{1}T_{1i}\cdot x_{i}+\gamma_{2}T_{2i}\cdot x_{i}+\gamma_{3}T_{3i}\cdot x_{i}+\gamma_{4}T_{4i}\cdot x_{i}+d_{t}+\epsilon_{i}, \end{array} \end{array}$$ (4)

which models the heterogeneity in the treatment effects by experimental conditions. Now the interaction terms *T*_*ji*_ ⋅ *x*_*i*_ refer to the interactions of individual treatment and baseline usage. Thus *γ*_*j*_ indicates how the individual treatment effect changes with the baseline water usage.

We report the estimation results of the two models in Table 4.

**Table 4:** Estimates: Heterogeneity analysis.

Estimates: Interaction term for TEs	Overall (Model (3))	By exp.conditions (Model (4))
Treatment (= 1)	11.47** (5.69)	
Treatment × baseline	−0.091*** (0.034)	
Campaign (*T*_1_ = 1)		14.11** (6.88)
Feedback (*T*_2_ = 1)		15.05** (6.75)
Rebate (*T*_3_ = 1)		12.1 (8.28)
Lucky draw (*T*_4_ = 1)		7.71 (6.14)
Campaign × baseline		−0.111** (0.043)
Feedback × baseline		−0.114*** (0.042)
Rebate × baseline		−0.095* (0.055)
Lucky draw × baseline		−0.066* (0.036)
Baseline	−0.02 (0.03)	−0.02(0.03)
Constant	5.67 (5.25)	5.67(5.25)
R squared	0.0619	0.0749
Observations	4495	4495

Note:

*, ** and *** indicate the significance level of 10%, 5% and 1% respectively.

It can be seen that there is strong interaction of treatments with baseline water usage. The coefficient of interaction effect is −0.091 for the overall treatment, significant at the 1% level. This means that a one-litre increase in baseline use leads to a reduction in water use by around 0.091 Litres. Thus, higher baseline households have a much higher conservation effect from the treatment. For example, an average household (of mean baseline usage 165 Litres per capita per day) has saved 5.915 Litres more water than that of a household with baseline LPCD of 100 L. Lower baseline households, however, seem to increase their water use after treatment.

The heterogeneity in treatment effect is of similar scale for all four experimental conditions. F-test shows that there is no significant difference in the interaction effects across the experimental conditions.

### 3.4 High baseline users’ behavior

**Fig 7:**
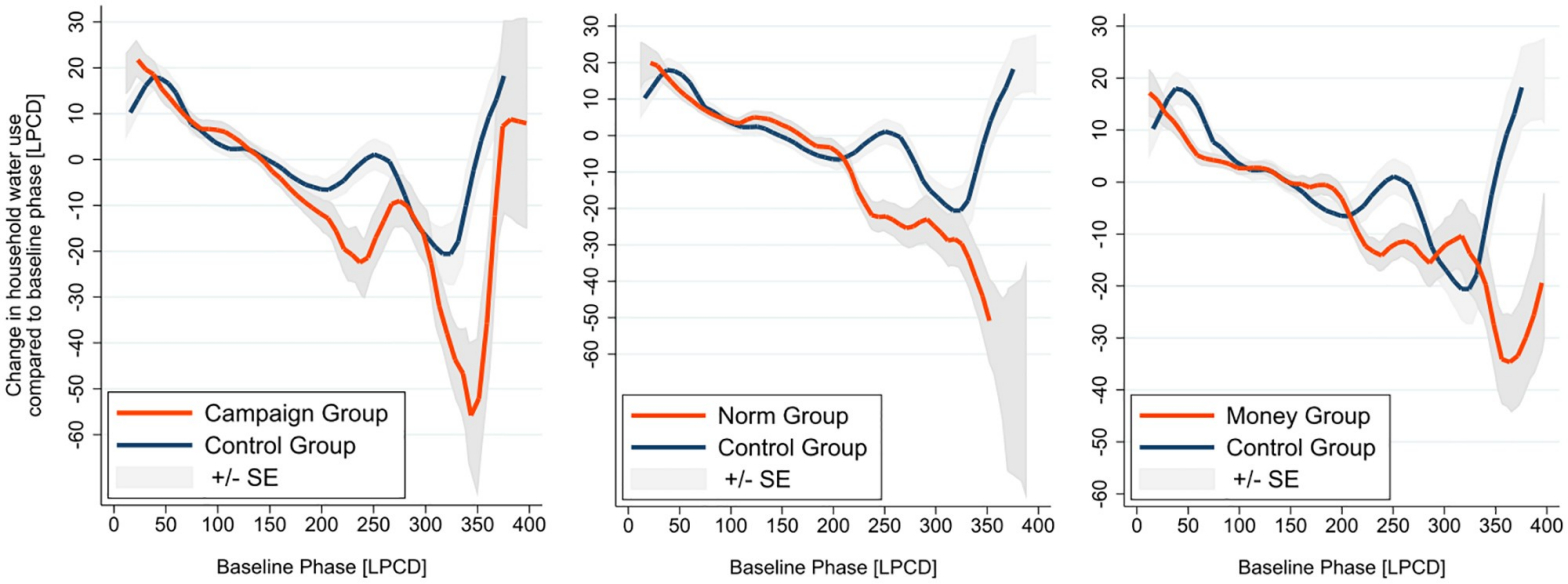
Heterogeneous treatment effects by different types of incentives.

We further investigate the behavior of the high baseline households. In particular, we aim to find out whether such a group respond to different interventions differently. Fig 7 presents the change in water use by households treated with different incentives compared to the control group: campaign group (*T*_1_), social norm and comparison group (*T*_2_) and monetary incentives group (*T*_3_ and *T*_4_). It shows that non-monetary incentives seem to have better effect.

To formally test the observation, we estimate the model

$$\begin{array}{c} \begin{array}{cc} \Delta_{y_{it}}=\alpha & +\beta_{bl}x_{i}+\beta_{1}T_{1i}+\beta_{2}T_{2i}+\beta_{3}T_{3i}+\beta_{4}T_{4i}\\ & +\eta_{1}T_{1i}\cdot H_{i}+\eta_{2}T_{2i}\cdot H_{i}+\eta_{3}T_{3i}\cdot H_{i}+\eta_{4}T_{4i}\cdot H_{i}+d_{t}+\epsilon_{i}, \end{array} \end{array}$$ (5)

where *H*_*i*_ is a dummy variable indicating whether household *i*’s baseline water consumption is higher (*H*_*i*_ = 1) or lower (*H*_*i*_ = 0) than the median level baseline water consumption. Now the interaction terms *T*_*ji*_ ⋅ *H*_*i*_ refer to the individual treatment for high baseline households compared to low baseline households. Thus *η*_*j*_ indicates how much more treatment effect that is resulting from high users.

The results are reported in Table 5. It can be shown that, high baseline households do save more water in response to the campaign and normative incentives. Compared to low baseline households which use slight more (around 1 litre), high users save 10.78 and 9.92 litres than their lower counterparts respectively, at the 5% significance level. However, with respect to the two monetary incentives, low users save some water (2.21 and 1.22 litres) and high users save a bit more (3.3 and 5.55 litres); but none of the effect is significant.

**Table 5:** Estimates for the high baseline group.

Estimates: Interactions with High Baseline Users (HIBL)	Model (5)
Campaign (*T*_1_ = 1)	1.04 (3.36)
Feedback (*T*_2_ = 1)	1.06 (3.14)
Rebate (*T*_3_ = 1)	−2.21 (3.00)
Lucky draw (*T*_4_ = 1)	−1.22 (3.09)
Campaign × HIBL	−10.78** (4.71)
Feedback × HIBL	−9.92** (4.72)
Rebate × HIBL	−3.3 (4.75)
Lucky draw × HIBL	−5.55 (5.06)
Baseline	−0.06*** (0.02)
Constant	13.79*** (3.75)
R squared	0.0683
Observations	4495

Note:

** and *** indicate the significance level of 5% and 1% respectively.

## 4 Discussion and conclusion

When informative, normative and monetary incentives are put together in a single field experiment, results show that the water conservation effect of the incentives are significant, despite no difference across treatments found. We thus believe that receiving periodic information with water saving tips, or such a ‘regular reminder’ plays a role in promoting household water conservation. This raises the awareness of the salience of water saving, as well as provides practical advice on how to save water at home.

However, given that our current setting relies on a feedback message which has to be consistent with the official information from the local utility, it is not sufficient to conclude that normative and monetary incentives have no additional effect. More evidence is needed to determine whether households would respond differently to such treatments. For example, more delicately customized feedback, or prize mechanisms should be designed to test and compare the effects.

In terms of heterogeneity, empirical results show that there is an average treatment effect of water saving around 4 Litre for all households, and 5.9 Litres for high baseline households. This finding is consistent with the literature and also implies the importance of policy tools targeting different groups of water users.

Furthermore, evidence also shows that the higher baseline households respond more positively to the campaign and normative incentives but less to the monetary incentives.

## Supporting information

S1 Fig First batch leaflets (notification round). (PDF)

S2 Fig Treatment round leaflets. (PDF)
