# Supplementary material for: Motivating household water conservation: A field experiment in Singapore
Source: PLoS One. 2019 Mar 20;14(3):e0211891. doi: 10.1371/journal.pone.0211891 (PMC6426227; doi:10.1371/journal.pone.0211891)
Supplement: S1 Fig — (PDF) [file pone.0211891.s001.pdf]

Back side

Water Saving Tips 省水小贴士

- **Take shorter showers**
  冲凉时，冲得快一些
- **Avoid running taps**
  用水时，水喉不要一直开住
- **Brush teeth with a cup**
  刷牙时，用水杯
- **Wash dishes with filled sink**
  洗碗时，用水池
- **Wash clothes with full load**
  洗衣时，把洗衣机装满
- **Choose water efficient facilities**
  购买时，选省水的洗衣机和水喉

Any query, feel free to call for information
如有疑问，欢迎致电
Tel: 6516 6725

Front side

Campaign

BLK 627 # 05-21 25 NOV 2016

USE WATER WISELY
上智若水

Every society shares the responsibility to promote sustainable use of water.
The future is in our hands.
水是宝贵的资源，每个社会成员都能为可持续发展尽一份力。
从家庭节水做起，共创美好未来！

EVERY DROP COUNTS
珍惜点滴 水 支持环保

All rights reserved by Institute of Water Policy, NUS 新加坡国立大学水政策研究所拥有最终解释权

Feedback

BLK 627 # 05-21 25 NOV 2016

Dear resident,
We will provide feedback on your household water consumption soon.
Try out some water saving tips today!

亲爱的居民，我们会反馈给您家庭用水情况。
现在开始尝试省水小贴士吧！

EVERY DROP COUNTS
珍惜点滴 水 支持环保

All rights reserved by Institute of Water Policy, NUS 新加坡国立大学水政策研究所拥有最终解释权

Rebate

BLK 627 # 05-21 25 NOV 2016

Dear resident,
We will provide feedback on your household water consumption soon.
If your water use is ***Efficient***, you will receive a $10 grocery voucher as prize!
Try out some water saving tips today!

亲爱的居民，我们会反馈给您家庭用水情况。如果达到**高效**，您将得到一份价值 $10 的购物礼券！现在开始尝试省水小贴士吧！

EVERY DROP COUNTS
珍惜点滴 水 支持环保

All rights reserved by Institute of Water Policy, NUS 新加坡国立大学水政策研究所拥有最终解释权

Lucky Draw

BLK 627 # 05-21 25 NOV 2016

Dear resident,
We will provide feedback on your household water consumption soon.
If your water use is ***Efficient***, you will stand to win a $200 grocery voucher in the lucky draw! Try out some water saving tips today!

亲爱的居民，我们会反馈给您家庭用水情况。如果达到**高效**，您就有机会抽奖赢得$200 的购物礼券！现在开始尝试省水小贴士吧！

EVERY DROP COUNTS
珍惜点滴 水 支持环保

All rights reserved by Institute of Water Policy, NUS 新加坡国立大学水政策研究所拥有最终解释权
