# Supplementary figures and images for: Motivating household water conservation: A field experiment in Singapore
Source: PLoS One. 2019 Mar 20;14(3):e0211891. doi: 10.1371/journal.pone.0211891 (PMC6426227; doi:10.1371/journal.pone.0211891)

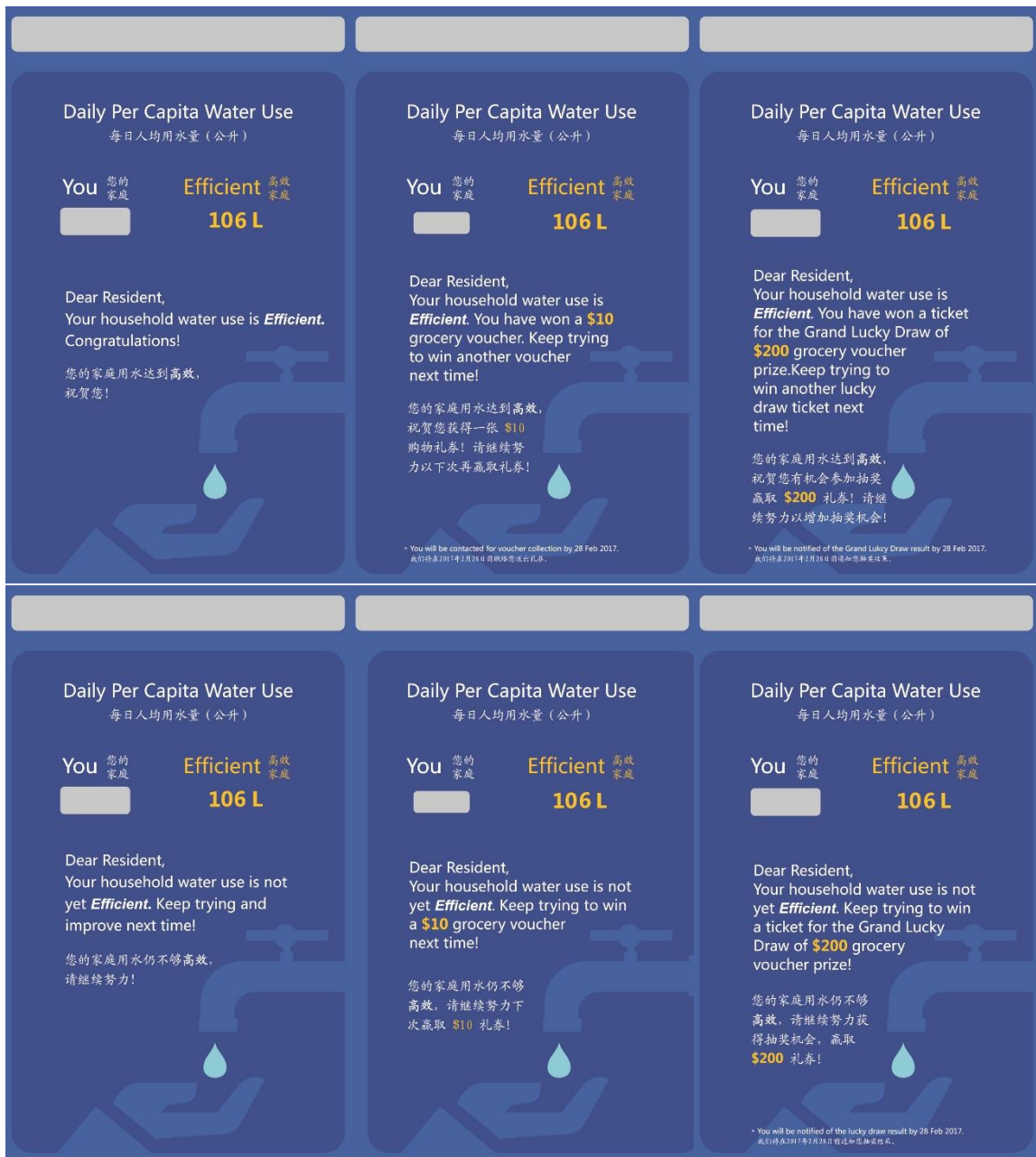

Supplement: S2 Fig — (PDF) [file pone.0211891.s002.pdf]
